# Riboflavin and Its Derivates as Potential Photosensitizers in the Photodynamic Treatment of Skin Cancers

**DOI:** 10.3390/cells12182304

**Published:** 2023-09-19

**Authors:** Małgorzata Insińska-Rak, Marek Sikorski, Agnieszka Wolnicka-Glubisz

**Affiliations:** 1 Faculty of Chemistry, Adam Mickiewicz University in Poznań, Uniwersytetu Poznańskiego 8, 61-614 Poznań, Poland; inka@amu.edu.pl (M.I.-R.); sikorski@amu.edu.pl (M.S.); 2 Department of Biophysics and Cancer Biology, Faculty of Biochemistry, Biophysics and Biotechnology, Jagiellonian University, Gronostajowa 7, 30-387 Kraków, Poland

**Keywords:** skin cancer, 3MeTARF, melanoma, riboflavin, lumichrome, singlet oxygen, PDT

## Abstract

Riboflavin, a water-soluble vitamin B2, possesses unique biological and physicochemical properties. Its photosensitizing properties make it suitable for various biological applications, such as pathogen inactivation and photodynamic therapy. However, the effectiveness of riboflavin as a photosensitizer is hindered by its degradation upon exposure to light. The review aims to highlight the significance of riboflavin and its derivatives as potential photosensitizers for use in photodynamic therapy. Additionally, a concise overview of photodynamic therapy and utilization of blue light in dermatology is provided, as well as the photochemistry and photobiophysics of riboflavin and its derivatives. Particular emphasis is given to the latest findings on the use of acetylated 3-methyltetraacetyl-riboflavin derivative (3MeTARF) in photodynamic therapy.

## 1. Introduction

Photodynamic therapy (PDT) is a treatment that uses a light-sensitive drug, oxygen, and a light source to destroy abnormal cells. Generally, PDT utilizes highly reactive oxygen species (ROS) to induce cell death in cancerous tissues. In addition to curing pathological changes of a neoplastic nature, it is also used in the treatment of other diseases. The application range of methods based on using light has been significantly extended by the mechanism of blood vessels closing, the treatment of diseases resulting from neovascularization, atherosclerosis, and restenosis after angioplasty procedures and in cases of arthritis. In addition, the method can be used in diagnostics, especially in the case of invisible lesions [1,2,3]. PDT, originally developed as an anti-cancer method, has revealed its expanded potential in light of numerous recent studies as a method of combating pathogenic microorganisms; it may be used in antimicrobial photodynamic therapy (aPDT), antifungal PDT, and antiparasitic PDT activities, as described in many reviews [4,5,6,7].

The effectiveness of the photodynamic therapeutic process depends on many factors, among which a few seem to be crucial: (1) selection of sensitizer, (2) the availability of oxygen in the tissue, (3) amount of singlet oxygen or the other ROS agents formed in the sensitization process, (4) the efficiency of light penetration through the disease-affected area or tumor, and (5) the sensitizer’s capacity for selective affinity to tumoral cells.

During therapy, the patient is treated with possibly nontoxic medicine that from photochemical point of view acts as a photosensitizer. These sensitizers accumulate in the diseased tissue. Pathological changes are then irradiated with the radiation of a wavelength lying in the absorption band of the sensitizer, which is activated via excitation to the singlet excited state. The relaxation of the excited state of the sensitizer molecule can occur through fluorescence, internal conversion, or through intersystem crossing, leading to the population of the triplet state. From the point of view of photodynamic therapy, the latter mechanism is the most desirable. Deactivation of the triplet state is a relatively long process (of the order of μs), thanks to which the sensitizer molecule that has reached this state can undergo further reactions. In the presence of oxygen, photosensitized reactions of oxidation may occur, and can proceed according to two mechanisms: electron transfer, resulting in the formation of radicals, superoxide anion (O_2_^•−^) or other oxidizing species (i.e., OH^•^, H_2_O_2_,) (type I); or energy transfer, leading to the formation of singlet oxygen (type II) [1,2,8,9]. In mechanism type I, an electron is transferred from the biological substrate to the photosensitizer, resulting in the oxidation of a biomolecule and the reduction of the photosensitizer to its radical anion Ps^•−^. As a result of a further reaction with molecular oxygen, a superoxide anion (O_2_^•−^) is formed, which can accumulate in the tissues. Under certain circumstances, an overproduction of superoxide anions may generate stronger agents, like the hydroperoxyl radical, HO_2_^•^, and the hydroxyl radical, HO^•^. Consequently, the cell’s redox regulatory system becomes perturbed, and more reactive oxygen species such as hydroxyl radicals or ONOO^−^ may initiate further reactions with biomolecules [10,11].

In mechanism type II, singlet oxygen directly oxidizes biomolecules. However, this mechanism requires a high concentration of oxygen in the tissue. It has been observed that the mechanism of photodynamic reaction may change during treatment. Although singlet oxygen formation predominates initially, as the oxygen concentration in the microenvironment decreases, the type I mechanism may prevail [1,2,12,13,14]. The concentration ratio of ^1^O_2_ and O_2_^•−^ formed simultaneously in both mechanisms depends on the local oxygen concentration, and ^1^O_2_ may be easily reduced to O_2_^•−^ [11].

Despite the fact that oxygen concentration differs in specific skin layers, it is believed that its content in the skin is high enough to generate photooxidation effect [11]. The formation of reactive oxygen species (ROS) necessary for cell destruction, is the source of both harmful and beneficial transformations in biological systems, especially those directly exposed to light and containing naturally occurring endogenous sensitizers. The intended use of this procedure has become the basis for effective therapeutic treatment.

A potential sensitizer should meet some photophysical requirements necessary for efficient action, such as a high molar absorption coefficient at the excitation wavelength, a high quantum yield of triplet state formation (Φ_T_), a long lifetime of the triplet state (τ_T_ > 1 μs), high energy of the triplet state (E_T_ ≥ 95 kJ mol^−1^), and high photostability [15]. It is also important to protect healthy tissues adjacent to pathological changes. Thus, appropriate photochemical properties such as non-toxicity, accumulation in a lesion, and selectivity of action are indispensable. Moreover, the short lifetime of singlet oxygen and the reactivity of the other oxidizing agents additionally indicate the necessity of their generation in near vicinity of the lesion.

However, in therapeutic aspects, it is not clear which of the oxidizing species is the main cytotoxic factor. One possible mechanism is the reaction based on the hydroxyl radical, which appears in tissues as the result of a dismutation reaction, enzymatic processes, or spontaneously. Another possible mechanism is the reaction between the superoxide anion and nitric oxide, which forms the highly toxic nitrite peroxide (ONOO^−^) [1,9].

The PDT method has some limitations related to possible side effects, such as long-term light sensitivity of the skin, excessive tissue damage in the treated area, and local disorders of the metabolism [2]. Therefore, when searching for and selecting a sensitizer, numerous factors should be taken into account, including its photosensitizing abilities and photostability. The photodynamic process may also be influenced by the light source and sensitizer concentration [8,16,17]. Sensitizers of hydrophilic properties, when incorporated into tissues, exhibit greater photostability than lipophilic ones. Photostability is reduced, when the photosensitizer molecule is bound to a protein molecule [8,18,19]. Sensitizers in the aggregated form show increased photostability compared to their monomeric form; however, when aggregated, they become inactive in the formation of singlet oxygen [8,16,17].

Although photobleaching of sensitizer can limit the effectiveness of therapy, this effect prevents damage to healthy tissues in the vicinity of those affected [8]. The process of the photodegradation of the sensitizer may determine its usefulness in PDT therapy, since after treatment the sensitizer, the products of its photodegradation should be safely removed from the organism or metabolized effectively [20]. Thus, pharmacokinetics also has important questions to answer.

## 2. Blue Light in PDT Therapy for Skin Cancer and Diseases 

Light is one of the most important factors used in photodynamic treatment, especially in energy carriers; it is necessary to excite the sensitizer and to prompt effective photodynamic action that is still safe for the patient and possibly harmless for the surrounding tissues. In a therapeutic context, it is important to find a compromise between the amount of energy carried by a light beam and its therapeutic potential [8,16,17].

As a non-invasive method, PDT requires light sources that can provide excitation energy directly to the lesion, which allows us to protect surrounding tissues. The light reaching the target tissue is also absorbed by the other compounds present there, mainly by hemoglobin, melanin or water. The “optical window” of living tissue extends between the absorption of heme and water, in the range of 600–1300 nm, with therapeutic range often referred as 600–1000 nm. Particularly desirable are sensitizers of intensive absorption bands in the red part of the spectrum, in the range of 600–800 nm [8].

The optimal wavelength emitted by the light source should be selected to obtain the maximum quantum yield of singlet oxygen (or other ROS), as well as the maximum depth of tissue penetration, in order to obtain the biological response.

Many types of light sources have been proposed for use in photodynamic therapy. Among them there are non-coherent emitters (halogen, xenon, fluorescent, LED diodes) and laser light sources that through optical fibers can provide radiation directly to the diseased tissue. The therapy’s effectiveness is also determined by the radiation dose and exposure time [8,16,17]. In vivo applications using a range of radiation should be minimized, especially those involving ultraviolet emission (due to the risk of mutagenesis) and infrared range (which causes overheating of the tissue) [21].

A significant turning point in therapies was the arrival of light-emitting diodes (LEDs), which are an alternative to commonly used lasers and lamps with optical filters. Some spectral ranges are still not available to traditional semiconductor lasers, while light-emitting diodes generate light in the UV, NIR and the entire visible band. Blue-emitting LEDs used for scientific purposes were developed in 1994 by Nakamura [22].

The practice of using blue light is widely applied in the treatment of *acne vulgaris*, associated with the colonization of the superficial layers of the skin by *Propionibacterium acnes*. These bacteria produce porphyrins, which, acting as sensitizer, induce photodynamic reactions, leading to cell death [23]. Therapy based on using blue light is highly effective in fighting bacteria families that colonize wounds, like *Staphylococcus aureus* and *Escherichia coli* [24], and bacteria causing periodontal diseases (*Staphylococcus epidermidis*, *Streptococcus pyogenes*) [25]. Blue radiation reduces the proliferation of keratinocytes and stimulates their differentiation, and when used in high doses, induces a cytotoxic effect on them. It has been shown that in vitro blue light diminishes the level of the cytokine IL-1α produced by leukocytes [26]. Although radiation known as blue light is a high-energy carrier (λ = 400–470 nm), its ability to penetrate through tissues is limited to a maximum depth of 0.3 to 2 mm [27,28]. On the other hand, its higher energy in comparison to that carried by red radiation has been appreciated in dermatology and cosmetology for the treatment of skin lesions that spread on the skin superficially [29]. Therefore, its application is dedicated to treat certain skin diseases, from *Actinic keratosis* and up to some types of cancer.

## 3. Photoreactivity of Flavin Derivatives: Photochemical Aspects

Riboflavin and its derivatives absorb light in UV-Vis region of electromagnetic spectrum. Their absorption spectrum partly covers the blue light region, being of special interest from the perspective of medical applications. Light absorption leads not only to photophysical, but also photochemical processes. Most riboflavin derivatives are quite susceptible to light-induced degradation, which may influence their sensitizing ability and determine their application in photodynamic therapy.

In terms of photochemistry, the irradiation of flavin derivatives by blue light leads to molecule degradation, with main photoproducts being lumichrome and lumiflavin in the case of riboflavin, or their derivatives of similar structure, respective to the studied flavin analogues [30,31,32,33,34,35]. Photoproduct distribution is also related to the conditions of the oxygen used in the experiment. Irradiation in the presence of oxygen leads to derivatives with successively fewer numbers of carbon atoms in a side chain; thus, there are more photoproducts detected in this reaction, while in the absence of oxygen, one main photoproduct is observed. This photoproduct is lumichrome [32,35] also known as the product of the metabolic reactions of riboflavin occurring in a cell.

Reaction occurs through light-initiated intramolecular processes with the involvement of the side chain. On the basis of some results, it was established that position 2 of the ribityl chain plays the crucial role [31,36] in a typical photodegradation process, according to the model proposed by some authors [37,38], via reduction, dealkilation and addition processes. Thus, the flavin molecule devoid of hydroxyl substituents in the exocyclic chain is unable to react, and photolysis leads to the other photoproducts [36]. The photodegradation processes are also suppressed when a flavin molecule is esterificated in a side chain [32].

**Figure 1:**
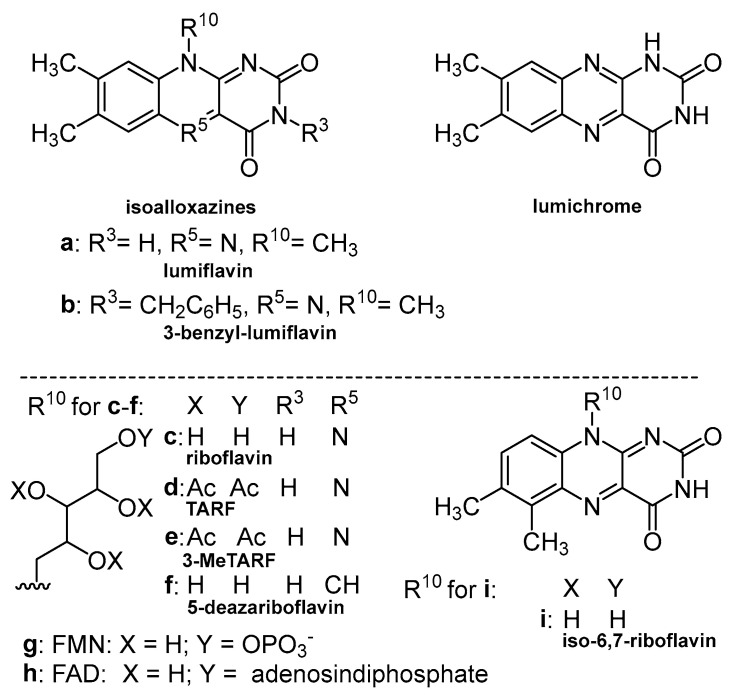
Structures of riboflavin and some of its derivatives.

The tetraacetyl analog of riboflavin (3MeTARF), see Figure 1, possesses the acetyl substituents in the ribose chain in its molecule, which inhibit the aforementioned intramolecular photoreactions [39,40].

The photolysis quantum yield of 3MeTARF (Φ_ph_ < 10^−5^ mol einst^−1^) is at least two orders of magnitude lower than that of riboflavin (Φ_ph_ = 1.1 × 10^−3^ mol einst^−1^) [32,41]. Thus, 3MeTARF (as well as TARF), similar to riboflavin in terms of spectral properties and singlet oxygen-generating ability (Table 1), shows higher stability after irradiation. Besides lower polarity, the photoresistance of this compound may be the reason for its enhanced photosensitizing abilities. (see paragraph 6, vide infra). In Figure 1, structures of some riboflavin analogues are presented, and Table 1 summarizes some photophysical properties important in PDT applications.

## 4. Photosensitization with Flavin Sensitizers: Biophysical Approach

In photophysical terms, flavins are considered sensitizers of singlet oxygen or other reactive oxygen species (ROS), and are useful in diverse photodynamic actions in medicine, photocatalysis, organic synthesis [56,57,58], environmental protection, cosmetology, and skin care products’ development [29,59]. The basis of riboflavins’ photosensitizing properties are their pH and redox properties [30], as well as their high quantum yield of intersystem crossing and the high oxidation potential of the triplet state [11].

The efficient triplet-state population of the sensitizer molecule, followed by the energy transfer reaction with oxygen molecules, results in the formation of an oxygen molecule in the singlet excited state, singlet oxygen (mechanism Type II), which is a strong oxidative agent. In the other mechanism, the sensitizer in the triplet state may react directly with the substrate present in the microenvironment, generating their radicals and ROS (i.e., superoxide anions, hydrogen peroxide, hydroxyl radicals), (mechanism Type I). These features, attributed also to flavins (both type I and II), predestine them to be potential factors in oxygen-mediated sensitization reactions [60,61,62,63]. Table 1 shows the fluorescence quantum yields (Φ_F_), intersystem crossing quantum yields (Φ_ISC_) and singlet oxygen generation yields (Φ_Δ_) of selected sensitizers. The long wavelength maxima indicate to what extent the absorption spectra of these compounds cover the blue light region.

It was found that flavins in the presence of oxygen can sensitize the oxidation of such substances as amino acids, proteins, nucleotides, lipids, and vitamins, which is a serious risk to living organisms. On one hand, this phenomenon can lead to damage to DNA and cell organelles, resulting in inflammation and accelerating the aging process [61,62]. In vivo photosensitization is believed to occur by electron transfer from DNA base molecules to riboflavin molecules. In such reactions, oxidized sensitizer free radicals are formed, which are the important transient species in the activation of reactive forms of procarcinogens and promutagens, and may attach to DNA molecules, causing irreversible changes in living organisms [60,61,62,64,65]. This phenomenon used intentionally can contribute to the fight against many harmful substances, microorganisms present in the environment, and serious diseases.

Flavins, at least riboflavin, FMN, and FAD, are non-toxic. Moreover, as water-soluble flavins are easily removable from the organism, which reduces the risk of potential side-effects. Additionally, excess riboflavin can be handled by organisms as they dispose of preventing mechanisms. In this role, the flavoprotein *dodecin* is thought to be a factor, which in living organisms causes ultra-fast quenching of riboflavin in the excited state and prevents uncontrolled light-induced reactions involving riboflavin as the sensitizer. An excess of riboflavin, bound by this protein, undergo degradation to lumichrome. The reaction occurs within the protein moiety and is harmless to the cell [66].

One of the main disadvantages in flavins’ application is their solubility, limited particularly in nonpolar solvents, which may negatively influence their bioavailability [67].

It has been demonstrated for flavoprotein molecules that the main role in initiating the photophysical cycle is attributed to the isoalloxazine chromophore, capable of efficiently populating the molecule’s lowest triplet state, while the protein environment increases the rate of intersystem crossing. This property is the basis for the proper functioning of the flavoproteins in nature, as was shown for the DNA repair processes occurring with FMN-based proteins’ involvement [68].

It was shown that mini singlet oxygen sensitizers (miniSOG) based on riboflavin-binding proteins can be considered attractive potential photosensitizers. The photoreactivity of these structures has been revealed for a series of ^1^O_2_ generators, (i.e., SOPP3), with a high singlet oxygen quantum yield, Φ_Δ_ = 0.61 [47]. In the light of the other study, the riboflavin chromophore was shown to be more effective (higher Φ_Δ_, see Table 1) in generating ^1^O_2_ than miniSOG based on its natural FMN chromophore structure, as the presence of the phosphate group is suggested to be one of the factors limiting oxygen’s access to the isolloxazine ring [45].

Photosensitization reactions might also be influenced by temperature [47], as it was shown in the temperature range 10–43 °C that the quenching rate constant of the triplet FMN by O_2_ molecules in the ground state increased 10 times, which leads to a higher rate of singlet oxygen formation under certain reaction conditions.

Flavins’ photochemical and photophysical properties depend on their various oxidation and protonation states, which is also reflected in the molecules’ conformation. Flavoenzymes achieve the desired biological effect via regulation of the flavin structure—either planar or bent—exchanging their reduced and oxidized forms [11]. Thus, this is one of the fundamental features to consider in searching for riboflavin analogues suitable for PDT applications [69,70]. Flavin molecules with a stable planar configuration have longer lifetimes compared to those with a bent “butterfly” structure [69,71]. It has been revealed recently that molecules with reduced flavins lose their planarity and thus become non-fluorescent [71,72,73]. In different protonation states, flavins, when bound to proteins, will show different behavior in the excited state. This was demonstrated for FAD, which in its neutral form undergoes very fast excited-state decay, and in its anionic form FAD_red_ displays an increased excited-state lifetime [74].

Lumichrome, which is the product of light-induced or enzymatic riboflavin degradation, is also known to be a good singlet oxygen generator [40,55]. Its absorption spectrum covers only a fragmentary blue light region, but it seems to be useful due to its high ^1^O_2_-generating parameters. This molecule, devoid of a ribityl chain, might be difficult to fasten to the protein molecule, but it has been suggested to provide better access to oxygen in the protein moiety to produce singlet oxygen [45].

## 5. Riboflavin and Its Derivates in PDT: Biological Issues

Recently, blue light in combination with photosensitizers such as curcumin [75,76], toluidine blue O [77], and also riboflavin-tryptophan (RT) gel [78] have been shown to treat skin with *acne vulgaris.* A comparative study of the treatment of acne lesions with RT gel or 13% ALA combined with blue diode irradiation showed good efficacy, not worse than ALA in the treatment of acne, with significant reductions in the number of acne lesions, severity, porphyrin, and sebum secretion. Side effects were minimal and well tolerated by all patients [78].

**Figure 2:**
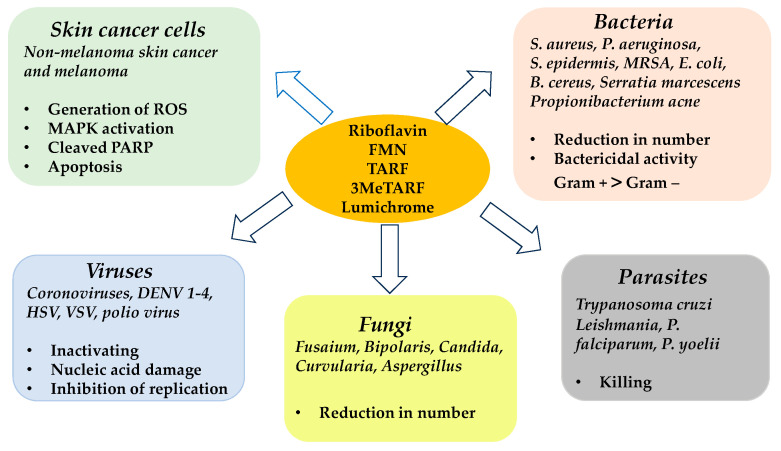
Diagram of the biological effects of riboflavin or its derivatives on pathogenic microorganisms or cancer cells after UVA or blue light activation [based on [34,43,84,85,86,87]].

Riboflavin has been shown to be a photosensitizer useful in the inactivation of some types of viruses and bacteria in blood products, while maintaining the activity and function of these products [79,80]. There is even a commercially available system that applies the UVA/riboflavin mechanism for pathogen inactivation in platelets and plasma during blood transfusions. In this system, UVA/riboflavin inhibits the replication of pathogens such as bacteria, fungi, parasites, viruses (see Figure 2) and donor leukocytes, while maintaining the homeostasis of erythrocytes, platelets, and plasma proteins [81,82,83]. The antimicrobial activity of riboflavin on its own and in combination with UVA is widely described in the review by Farah et al. [84].

The role of riboflavin itself was also indicated in the treatment of hematogenous metastasis [88]. However, extensive use of UVA light (315/320–400 nm) poses a risk of photoaging, skin pigmentation, and even skin cancer. This argument significantly supports the application of blue light for therapeutic purposes.

Photophysical properties, such as blue light absorption, efficient intersystem crossing, and the long lifetime of the triplet state, make flavins useful as potential therapeutic agents in skin diseases including skin cancer. In addition, riboflavin has been proposed as an adjuvant in the treatment of skin cancer with cisplatin [89], and as a photosensitizer in the treatment of cervical cancer cells (HeLa) [90].

There are two main forms of skin cancer: non-melanoma and melanoma. Non-melanoma skin cancer is an out-of-control growth of abnormal epidermal cells (keratinocytes) or cells of epidermal origin (Merkel cells). In contrast, melanoma originating from abnormal melanocytes (of neural crest origin) is one of the most aggressive and deadly forms of cancer due to its ability to metastasize and acquire resistance to chemotherapy [91]. The main types of non-melanoma skin cancer are basal cell carcinoma (BCC), squamous cell carcinoma (SCC), and Merkel cell carcinoma (MCC) [92], while melanoma include superficial spreading melanoma (SSM), lentigo maligna melanoma (LMM), and nodular melanoma (NM) [91].

However, since localized cutaneous melanoma is a skin tumor, which is easy to irradiate, in its early stages (<1 mm thickness), PDT is still one of the promising therapeutic procedures. Indeed, flavin mononucleotide (FMN) in combination with blue light has a destructive effect on human melanoma cells (Mel MTP, Mel IL, Mel Z, A375), as well as mouse melanoma cells (M-3, B16 F10) (Ex 438 nm) [86] and squamous cell carcinoma (SCC-13) (Ex 446 nm) [93]. Moreover. skin cancer cells are effective against human colorectal carcinoma cells (HCT116 tumor cells) [88].

In addition, the efficacy of FMN-induced PDT in vivo was confirmed on xenografts formed from A375 cells and Mel IL. Melanoma xenograft regression was observed in mice (85–90% volume in 50 days) after systemic intravenous injection of FMN, followed by blue light irradiation (450 nm, 20 J/cm^2^) for 15 min. Interestingly, for this model, the reduction in the growth rate of distant tumors to control tumors was estimated to be a 20% inhibition for the single-PDT procedure and 30% for the double-PDT procedure [88]. FMN is generally recognized as safe and practically non-toxic, at least up to 10 g/kg via oral administration to rats [94]. Moreover, being a cofactor for a variety of flavoprotein enzyme reactions, FMN is not recognized by human ABC transporters as an exogenous drug, so FMN can overcome the drug resistance often occurring in case of tumors [88].

Recent studies have indicated that riboflavin derivatives can act more effectively than riboflavin itself, after excitation with blue light. Such an example is TARF, which inhibits cell proliferation more effectively than riboflavin [93]. The water-soluble riboflavin molecule enters the cell via the riboflavin carrier protein (RCP) present in the cell membrane. It appears that the amount of RCP in some cancers, including human squamous cell carcinoma (SCC), as well as in A431 cells, an in vitro tissue model of SCC is significantly higher than in healthy skin. Riboflavin is taken up primarily by endocytosis and is widely translocated to mitochondria [95]. Bartmann et al. showed that A431 cells incubated for 5 min in RPMI1640 medium containing an excess of riboflavin (1 mM) accumulate approximately 53.5% of this substance, which increases linearly to 87.1% after 30 min of incubation [95]. Standard RPMI1640 medium, according to the manufacturer, contains 2 to 20 mg/L riboflavin, or 5–53 µM, which is 20–200 times less than in the quoted experiment. In general, there is no evidence of riboflavin toxicity in humans due to its excessive intake, as its absorption becomes less efficient as the dose increases. There are no adverse effects from high riboflavin intake from foods or supplements up to 400 mg daily for at least three months [96]. Cellular uptake of 3MeTARF remains unknown, but since it is less polar than Rfl, it can be speculated that it binds or interacts with membranes. The lower polarity of this molecule may also increase its bioavailability as a potential sensitizer [97].

In fact, 3MeTARF induces oxidative stress and cell death in skin cancer (A431) and melanoma cells (WM115) [34,85], and also kills the parasite *Leishmania major* more effectively than riboflavin [43]. Interestingly, lumichrome, the product of light-induced or enzymatic degradation of riboflavin, appeared to be selective towards bacteria, being ten times more effective against Gram-positive bacteria than against Gram-negative bacteria [87]. Recently, Crocker et al. [98] showed that the use of LED light (1 × 10^5^ lux) and flavin with a bromine atom in the structure, increases the production of singlet oxygen through a heavy-atom effect, resulting in an increased PDT effect against both bacteria and viruses. This inspiring research may result in the design of a new generation of flavins.

## 6. Apoptosis as a Mechanism of 3MeTARF-Induced Melanoma and Non-Melanoma Skin Cancer Cell Death

Uncontrolled growth, angiogenesis, and avoidance of apoptosis are characteristic features of all cancer cells, regardless of their type or cause of occurrence [99]. Apoptosis, a natural mechanism of genetically programmed cell death regulated by caspases, is one of the processes of cancer prevention. Activation of apoptotic caspases results in the inactivation or activation of substrates and the initiation of a cascade of signaling events, permitting the controlled breakdown of intracellular components while avoiding inflammation of and damage to surrounding cells. Based on the adaptors and initiator caspases involved (caspase 8, -9), different apoptotic pathways (extrinsic or intrinsic) can be distinguished. However, both of them consequently lead to the activation of effector caspases (caspase 3, -6, -7), which are responsible for the final death of the cell in the so-called executive phase of apoptosis [100]. They differ in the initial stages of the pathway. The intrinsic pathway occurs when proapoptotic proteins of the BCL-2 family cause permeabilization of the outer mitochondrial membrane, leading to a decrease in membrane potential, an increase in membrane permeability, and the release of cytochrome c. This activates caspase 9 and Apaf-1. The extrinsic pathway occurs when death receptors on the cell surface destined for death, such as Fas, are engaged by their ligands. The binding of the ligand to the receptors on the target cell results in aggregation of the receptors on the cell surface. This aggregation recruits adaptor proteins at the cytoplasmic site of the receptors, forming a death-inducing signaling complex (DISC). DISC formation brings procaspase molecules closer together, facilitating their autocatalytic activation and release into the cytoplasm, where they activate the caspase cascade. Active caspase-8 also mediates the cleavage of the proapoptotic protein, BID, which then releases mitochondrial proapoptotic factors linking the two pathways [101].

The expression levels of pro-apoptotic proteins BAX, but not the anti-apoptotic protein BCL-2, were markedly increased in lymphocytes after riboflavin and UVA [102]. The mechanism of 3MeTARF-induced apoptosis remain unknown; however, we suppose that since it generates oxidative stress, it works according to the intrinsic pathway, similarly to riboflavin and TARF [93].

Loss of apoptotic control due to inhibition of caspase function, or an increase in anti-apoptotic BCL-2 proteins and loss of pro-apoptotic BAX and/or BAK, allows cancer cells to survive longer and to accumulate mutations that deregulate cell proliferation and interfere with their differentiation, stimulate angiogenesis, and increase invasiveness during tumor progression [103,104]. The induction of apoptosis in cancer cells is the goal of many therapies, including PDT. Apoptosis, unlike necrosis, eliminates cancer cells and also those infected by viruses without causing inflammation; therefore, it is a desirable process.

Our recent in vitro studies confirmed that 3MeTARF induces apoptosis of WM115 melanoma and A431 human epidermal cancer cells under blue light, while the percentage of necrotic cells did not exceed 6% [34,85].

**Figure 3:**
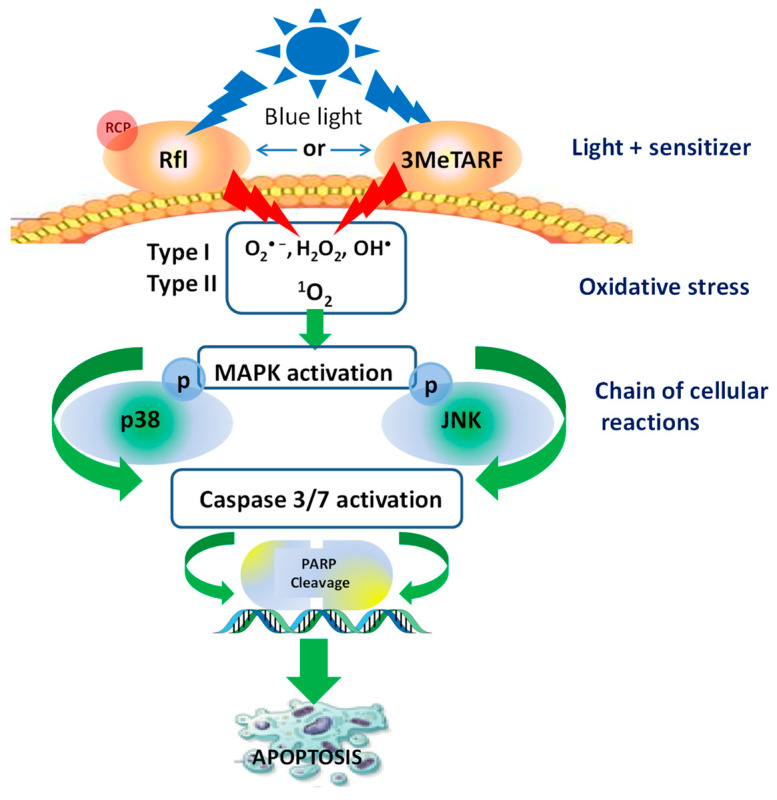
Scheme of riboflavin and 3MeTARF action in tumor cells upon blue light activation. RCP: riboflavin-binding protein; p: phosphorylation [based on [34,85]].

Mechanistically, irradiation of melanoma cells as well as skin cancer cells with blue LED light (438 nm) in the presence of 3MeTARF generates reactive oxygen species and increases intracellular oxidative stress. Next, oxidative stress, photogenerated in these reactions, activates MAPK by increasing phosphorylation of p38 and JNK, leading to caspase 3 and caspase 7 activation, PARP cleavage, and apoptosis (see Figure 3).

It has been demonstrated that pre-incubation of A431 cells with inhibitors specific for protein phosphorylation of p38 and JNK (SB203580, SP600125, respectively) results in decreased caspase 3/7 activation and PARP cleavage [34,85]. Since the phototoxic effect of 3MeTARF was five to ten times more pronounced, when compared to riboflavin, it again support the hypothesis that riboflavin’s analogue 3MeTARF may be more useful and desirable as an agent for use in photodynamic oxidation processes, including its therapeutic potential. The articles [34,85] also note the lack of toxicity in vitro of flavin in the dark at the concentrations used, of riboflavin up to 50 µM, and of 3MeTARF up to 10 µM. At present, it remains unknown if 3MeTARF in higher concentrations in the dark is nontoxic. Moreover, the metabolism of 3MeTARF and its effects in vivo is under-researched, and requires further study.

## 7. Conclusions

The photosensitizing properties of dyes depend on their chemical structure, physicochemical properties, and their ability to penetrate and be retained in diseased tissue. However, it is difficult to find dyes that simultaneously meet all the requirements set for them.

Riboflavin is described as a good sensitizer, but its efficiency is limited due to its susceptibility to photodecomposition (vide supra) [39,44,105]. Flavins’ poor solubility and bioavailability may also present challenges in finding derivatives with relevant properties among them. The application of blue light, which is required for the activation of flavin derivatives, might be a significant advantage when using them in the photodynamic treatment of superficially located lesions or for other photodynamic purposes that do not require deep light penetration. Due to extensive achievements in synthesizing new flavin analogues and better understanding of their photosensitizing activity, there are now real opportunities to facilitate the identification of the derivatives with the best potential in PDT [36]. Thus, it seems to be worth searching for flavin analogues that will allow us to avoid riboflavin’s disadvantages while retaining its favorable qualities, mainly its high quantum yield of singlet oxygen generation and non-toxicity [84,106,107].

Further studies using in vivo models on its mechanisms of action and effectiveness will determine the utility of flavin derivatives in various PDT therapies.

## Figures and Tables

**Table 1:** Flavin-based photosensitizers activated upon blue light excitation.

Sensitizer	λ_A_ [nm]	λ_F_ [nm]	Φ_F_	τ_F_ [ns]	Φ_ISC_	Φ_Δ_
Riboflavin^MeOH [a]^	444	532	0.39	6.4/5.4 ^[k]^	0.6 ^[l]^	0.51
Riboflavin^water [b]^	444	537	0.28	5.1	0.7 ^[m]^	0.48 ^[l]^
TARF^MeOH [c]^	446	525	0.46	-	-	-
TARF^water [b]^	446	-	-	-		0.66
3MeTARF^MeOH [d]^	448	513	0.089/0.12 ^[c]^	5.4	-	0.61/0.46 ^[e]^
3MeTARF^water [d]^	451	520	0.11	4.4	-	-
3MeTARF^PBS/DMSO [e]^	450	-	-	-	-	0.49
FMN^TRIS(d) [f]^	-	-	-	-	-	0.57
FMN^water [g]^	-	-	0.23 ^[n]^	5.1 ^[n]^	-	0.59
miniSOG [FMN]^TRIS(d) [f]^	-	-	0.4	5.5	0.6	0.04
miniSOG [Rfl]^TRIS(d) [f]^	-	-	-	-	-	0.10
SOPP3 ^[h]^	-	-	-	-	-	0.61^D^_2_^O^
Iso-6,7-riboflavin^MeOH [a]^	447	552	0.20	4.2	-	0.70
5-deazariboflavin^MeOH [i]^	400	455	0.11	3.98	-	0.33
Lumichrome^MeOH [j]^	384	453	0.032	1.04 ^[o]^	-	0.85
Lumichrome^water [j]^	385	479	0.088	2.7	0.63 ^[b]^/0.69 ^[p]^	0.36^D^_2_^O [p]^
[Ref.]: [a] [42]; [b] [40]; [c] [43]; [d] [44]; [e] [34]; [f] [45]; [g] [46]; [h] [47]; [i] [48]; [j] [49]; [k] [50]; [l] [51]; [m] [52]; [n] [53]; [o] [54]; [p] [55].						

λ_A_—long-wavelength absorption maximum, λ_F_—fluorescence maximum, Φ_F_—fluorescence quantum yield, τ_F_—fluorescence lifetime, Φ_ISC_—intersystem-crossing quantum yield and Φ_Δ_—singlet oxygen formation quantum yield.

## Data Availability

Not applicable.
